# Surface Modification of Polydopamine Particles with Polyethyleneimine Brushes for Enhanced Stability and Reduced Fragmentation

**DOI:** 10.3390/polym17091209

**Published:** 2025-04-28

**Authors:** Su Hyeon Son, Eun Jin Kim, Hye Young Koo, Won San Choi

**Affiliations:** 1 Department of Chemical and Biological Engineering, Hanbat National University, 125 Dongseodaero, Yuseong-gu, Daejeon 305-719, Republic of Korea; shujh19@naver.com (S.H.S.); kimej106@naver.com (E.J.K.); 2 Functional Composite Materials Research Center, Institute of Advanced Composite Materials, Korea Institute of Science and Technology (KIST), 92 Chudong-ro, Bongdong-eup, Wanju-gun 55324, Republic of Korea; koohy@kist.re.kr

**Keywords:** polydopamine, stability, fragmentation, surface engineering

## Abstract

Polydopamine (Pdop) particles possess unique properties but suffer from inherent instability in aqueous environments due to the gradual release of Pdop fragments. This study demonstrated the successful enhancement of the stability and reduction in fragmentation in Pdop particles through surface engineering strategies. Specifically, we investigated the effects of polyelectrolyte multilayer (PEM) coating and polyelectrolyte (PE) brush grafting. Our results showed that PE brush grafting, particularly with long-chain polyethyleneimine (PEI), was more effective in suppressing Pdop fragment release compared to PEM coating. The L-PEI grafted Pdop particles (2.28 chains/nm^2^) exhibited remarkable stability across a wide pH range (3–9), with inhibition rates exceeding 90% in most cases, reaching 93% at pH 5. Furthermore, a direct correlation between PEI grafting density (0.64 to 2.28 chains/nm^2^) and inhibition rate was observed, with higher densities yielding greater stability. These findings offer a promising approach for stabilizing Pdop particles for diverse applications.

## 1. Introduction

Polydopamine (Pdop) have garnered significant attention in various fields due to their unique properties such as strong adhesion, biocompatibility, and tunable surface chemistry [1]. These characteristics have led to their widespread application in numerous fields, including biomedicine, catalysis, energy, military, and materials science [2,3]. Dopamine in alkaline conditions undergoes oxidation, followed by cyclization and rearrangement to form indoloquinone [1,2,3]. Subsequently, indoloquinone reacts with catechol groups present in other dopamine molecules, leading to the formation of Pdop. Pdop is a water-insoluble biopolymer, appearing as a brown–black substance. The Pdop structure consists primarily of indole units with varying degrees of hydrogenation, interconnected via carbon–carbon bonds between their benzene rings. Given its versatile synthesis, both with and without templates, Pdop has been utilized to enhance the oil/water separation efficiency [4,5,6,7], adsorption capacity [8,9,10], catalytic efficiency [11,12,13,14], and stability [15,16,17,18,19,20] of composite materials across various applications. Active functional groups can be introduced onto the Pdop surface through Michael addition or Schiff base formation, enabling the creation of novel functional materials [21,22,23]. Furthermore, Pdop’s strong near-infrared (NIR) absorption presents significant potential for applications, including photothermal therapy [24], wastewater treatment [25], desalination [26], and photocatalysis [27,28,29,30]. However, a critical limitation of Pdop films or particles is their instability in aqueous solutions. Over time, Pdop particles tend to undergo degradation, resulting in the detachment of smaller fragments from the particle surface [3,31]. Previously, Pdop was primarily utilized under neutral conditions, thus the stability issues of Pdop were not significantly emphasized [7,8,9,10,12]. However, the release characteristics of Pdop fragments from Pdop particles become evident under acidic and basic conditions [3,31], underscoring the necessity for research to ensure long-term stability across diverse environmental conditions. This phenomenon not only compromises the structural integrity of the particles but also limits their long-term stability and performance in various applications. The release of Pdop fragments has hindered the accurate performance evaluation of Pdop-based catalysts and ultimately leads to their degradation [11]. The degradation of Pdop particles in aqueous media is particularly problematic in biomedical applications, where the release of small fragments can induce adverse biological responses. The Chen group reported that the degradation products of Pdop possess biological activity, specifically demonstrating an anti-inflammatory effect on macrophages. This finding highlights that released Pdop fragments are not inert and can indeed interact with biological systems [32]. Furthermore, the release of Pdop fragments can significantly interfere with accurate performance evaluation in many studies.

In this study, we propose to enhance the stability and reduce the fragmentation of Pdop particles by employing several surface engineering strategies. Specifically, we will utilize polyelectrolyte multilayer (PEM)-coating and polyelectrolyte (PE) brush-grafting approaches. We anticipate that these modifications will significantly improve the stability of Pdop particles, thereby expanding their potential applications in various fields, including surface coatings, desalination, drug delivery, and tissue engineering.

## 2. Experimental Section

### 2.1. Materials

Dopamine hydrochloride (100%), tris(hydroxymethyl)aminomethane (≥99.8%), long-chain polyethyleneimine (L-PEI, MW = 750,000, 50 wt % in H_2_O) and short-chain polyethyleneimine (S-PEI, MW = 25,000, ≤1 wt % in H_2_O) solutions, ethanol (EtOH, 96.5%), ammonium hydroxide solution (NH_4_OH, 28−30%), sodium polystyrene sulfonate (PSS, MW = 70,000), and poly(allylamine hydrochloride) (PAH, MW = 17,500) were purchased from Sigma-Aldrich. All chemicals were used as received without further purification. Deionized (DI) water with a resistivity of 18.2 MΩ/cm was obtained from a Millipore Simplicity 185 system.

### 2.2. Synthesis of Pdop Particles

Polydopamine (Pdop) particles were synthesized following a modified literature procedure [12,15,31]. A mixture of 27 mL of deionized (DI) water, 12 mL of ethanol (EtOH), and 120 μL of ammonium hydroxide solution (NH_4_OH) was prepared and stirred at room temperature for 30 min. An aqueous solution of dopamine hydrochloride (150 mg/3 mL) was then slowly added dropwise to the mixture. The resulting solution was reacted for 30 h, during which time the pH was maintained between 8.4 and 8.6. The resulting precipitate was collected and washed three times with DI water.

### 2.3. Synthesis of Pdop/(PEI/PSS)_3_ and Pdop/(PAH/PSS)_3_ Multilayers

PEMs were assembled onto the Pdop particles using a layer-by-layer deposition technique. For the Pdop/(PEI/PSS)_3_ system, 2 mL of a PEI solution (2 mg/mL) was first added to an aqueous suspension of Pdop particles (10 mg). The dispersion was vigorously agitated on a shaking apparatus for 20 min to allow for adsorption of the PEI. The resulting particles (Pdop/PEI) were then collected by centrifugation and rinsed three times with deionized water to remove any unbound PEI. The collected particles were subsequently redispersed in 2 mL of a PSS solution (2 mg/mL) and agitated for another 20 min to form Pdop/(PEI/PSS) particles. This adsorption and rinsing process was repeated three times to create the desired Pdop/(PEI/PSS)_3_ multilayer structure. The Pdop/(PAH/PSS)_3_ multilayer system was synthesized using the same layer-by-layer deposition method, substituting PAH for PEI in the initial adsorption step.

### 2.4. Synthesis of Pdop/L-PEI or S-PEI Brushes

The grafting of the PEI brush onto the Pdop particles was performed following the established method detailed in our previous work [8,11]. Pdop particles (10 mg) were added to 40 mL of a solution containing L-PEI (12 mg) and Tris-HCl buffer (48.5 mg). The resulting mixture was reacted under reflux at 60 °C for 3 h to graft the L-PEI brushes onto the Pdop particles using a “grafting-to” method. After 3 h, the reaction mixture was cooled to room temperature, and the resulting particles were collected by centrifugation and washed three times with deionized water to remove any unbound PEI. The collected Pdop/L-PEI brush particles were then dried in an oven at 50 °C. The same procedure was followed for the synthesis of Pdop/S-PEI brushes, substituting S-PEI for L-PEI. The L-PEI brushes with low and high grafting densities were prepared using L-PEI at concentrations of 2.4 mg and 36 mg, respectively. These concentrations represent 1/5 and 3 times the concentration of L-PEI used for the medium grafting density.

### 2.5. Characterization

Field-emission scanning electron microscopy (FE-SEM) was performed using a Hitachi S-4800 microscope (Hitachi High-Technologies Corporation, Tokyo, Japan). Fourier transform infrared (FT-IR) spectra were acquired on a Thermo Scientific Nicolet iS5 spectrometer (Thermo Fisher Scientific, Madison, WI, USA). Zeta potential measurements were conducted using a Malvern Zetasizer Nano ZS (Malvern Panalytical Ltd., Malvern, Worcestershire, UK) at room temperature, with the reported values representing the average of three independent measurements. Particle size distributions were analyzed using a particle size analyzer (Beckman Coulter UPA-150, Brea, CA, USA). UV–Vis absorption spectra were recorded on a Thermo Scientific Evolution 201 spectrophotometer (Thermo Fisher Scientific, Madison, WI, USA). All experiments were performed at least four times, and the data are presented as mean ± standard deviation.

## 3. Results and Discussion

**Figure 1:**
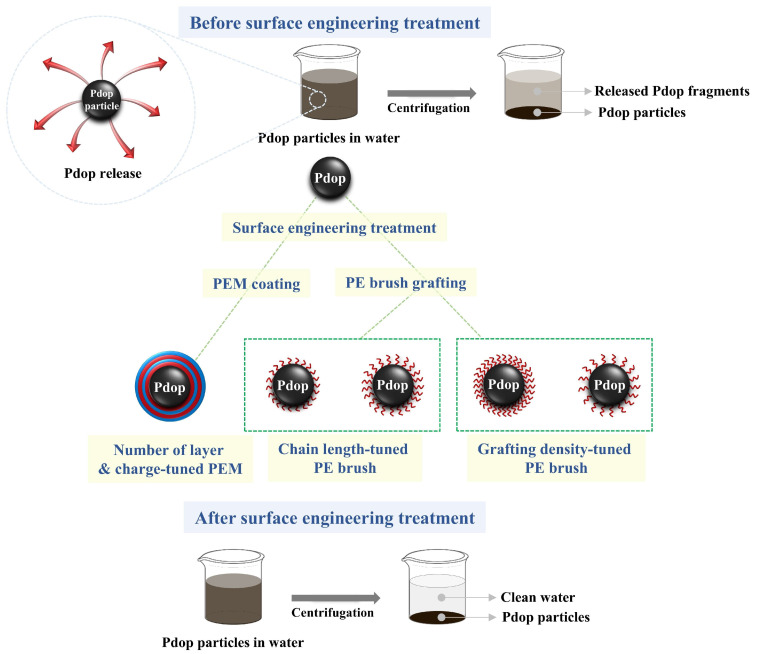
Schematic illustration of surface engineering strategies employed to enhance the stability and mitigate fragmentation of Pdop particles: (1) PEM coating and (2) PE brush grafting.

Figure 1 shows several surface engineering strategies aimed at enhancing the stability and mitigating fragmentation of Pdop particles. Pdop particles undergo gradual release of fragments into aqueous solutions over time. To address this challenge, two primary approaches including PEM coating and PE brush grafting were employed. First, PEMs were formed on the surface of Pdop particles through a layer-by-layer self-assembly process. The influence of key parameters, including the number of coating layers and the charge of the PEs, on the stability of Pdop particles was investigated. Second, PE brushes were grafted onto the surface of Pdop particles. The effects of varying PE chain length and grafting density on the stability of Pdop particles were investigated. Surface engineering strategies can effectively improve the stability of Pdop particles.

**Figure 2:**
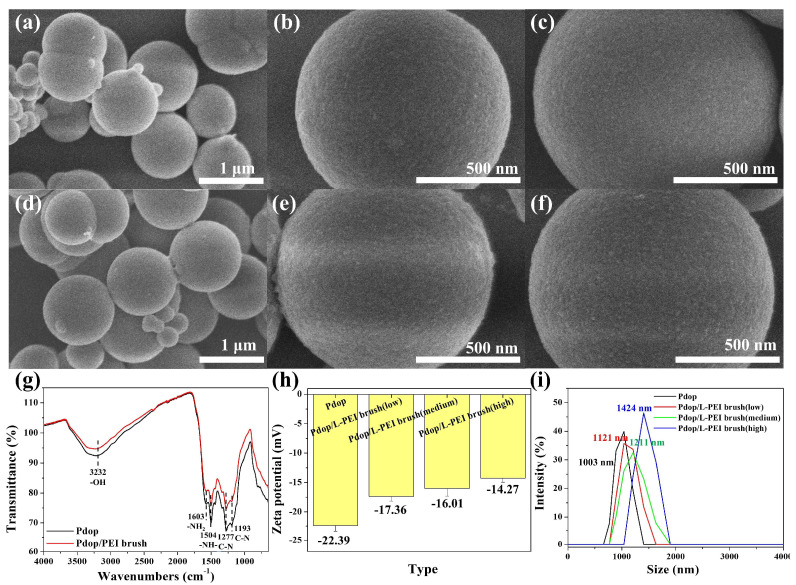
Characterization of Pdop particles and Pdop/PEI brush particles. SEM images of Pdop particles before and after grafting with PEI brushes: (**a**–**c**) Pdop particles and (**d**–**f**) Pdop/PEI brush particles. (**g**) FT-IR spectra of bare Pdop particles and Pdop/PEI brush particles. (**h**) Zeta-potential and (**i**) DLS data for Pdop/PEI brush particles with varying grafting densities (low, medium, and high).

Scanning electron microscopy (SEM) images revealed that Pdop particles, with an average size of 1010 nm, exhibited a smooth surface morphology (Figure 2a–c). No significant morphological differences were observed between bare Pdop and Pdop/PEI brush particles (Figure 2a–f). FT-IR spectroscopy was employed to verify the formation of Pdop and the successful grafting of PEI brushes. The successful synthesis of Pdop particles was confirmed by the presence of characteristic absorption peaks at 3232 cm^−1^ (-OH stretching), 1603 cm^−1^ (-NH_2_ bending), 1504 cm^−1^ (-NH- scissoring), 1277 cm^−1^ (-CN bending), and 1193 cm^−1^ (-CN stretching (black line, Figure 2g) [12,31]. While an overall decrease in peak intensity was observed after PEI brush grafting, no significant shifts or the appearance of new peaks were detected due to the spectral similarity between Pdop and PEI (red line, Figure 2g). However, for the Pdop/PEI brush, successful PEI grafting onto Pdop was confirmed by a change in the -NH_2_/-NH- ratio (from 1.05 to 1.1) at 1603 cm^−1^ (-NH_2_ bending) and 1504 cm^−1^ (-NH- scissoring) (red line) [31]. FT-IR data supporting the formation of the Pdop/(PEI/PSS)_3_ and Pdop/(PAH/PSS)_3_ systems can be found in the Figure S1. The 1033 cm⁻^1^ peak is attributed to the inherent symmetric stretching mode of the -SO_3_ group in PSS, whereas the 1007 cm⁻^1^ (or 1006 cm⁻^1^) peak potentially signifies alterations resulting from interactions with PEI (or PAH). This peak’s observation may be indicative of the establishment of ionic linkages between PSS and PEI (or PAH) (Figure S1). Zeta potential and dynamic light scattering (DLS) measurements were performed to investigate the characteristics of the Pdop/PEI brush. The results confirmed the successful grafting of PEI brushes with varying grafting densities onto the Pdop particles. Before PEI grafting, the surface charge of the Pdop particles at pH 6.8 was −22.39 mV, likely attributed to the presence of catechol groups (Figure 2h). This value is consistent with typical zeta potentials (−20 to −30 mV) observed for Pdop particles at pH 6–7 [33,34], although surface catechol group density can influence the measured zeta potential. Following PEI grafting with low, medium, and high grafting densities, the surface charge of the resulting particles shifted to −17.36 mV, −16.01 mV, and −14.27 mV, respectively (Figure 2h). This positive shift in surface charge is indicative of successful PEI grafting, as the positively charged PEI moieties imparted a more positive surface charge to the Pdop particles. The formation of the PEM coating onto Pdop particles was confirmed by Zeta potential measurements. Figure S2 depicts the Zeta potential values recorded after the sequential adsorption of PEs for the Pdop/(PEI/PSS)_3_ and Pdop/(PAH/PSS)_3_ systems. A clear alternation between negative and positive Zeta potential values was observed, indicating the layer-by-layer self-assembly of the PEM. These results strongly suggest the successful formation of the (PEI/PSS)_3_ and (PAH/PSS)_3_ PEM systems onto the surface of the Pdop particles.

The hydrodynamic radius of the Pdop particles in aqueous solution was measured to be 1003 nm (Figure 2i). Following PEI grafting with low, medium, and high grafting densities, the particle sizes increased to 1121 nm, 1211 nm, and 1424 nm, respectively. These increases in particle size are consistent with the successful grafting of PEI brushes with estimated lengths of 118 nm, 208 nm, and 421 nm onto the surface of the Pdop particles. Although the PEI chain length was consistent, the size of the resulting particles (Pdop/PEI brush) varied depending on the grafting density. As grafting density increases, the PEI brushes are forced closer together, enhancing electrostatic repulsion and stretching, which in turn increases the effective length of the PEI chains [35].

**Figure 3:**
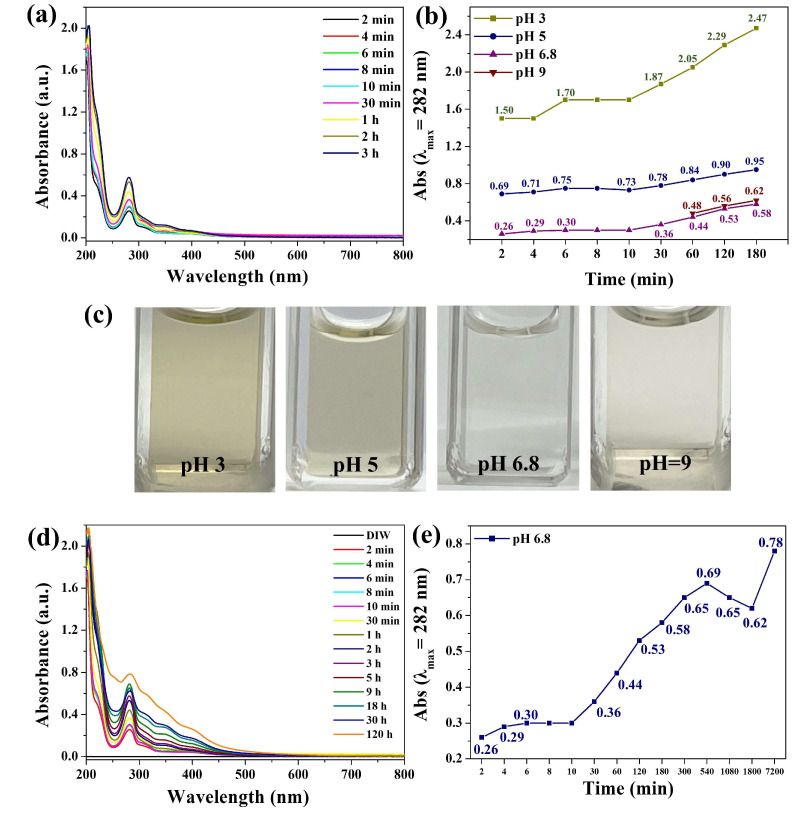
Analysis of Pdop fragment release from Pdop particles under varying conditions. (**a**) UV–Vis absorption spectra of Pdop fragments released from Pdop particles at pH 6.8 as a function of time. (**b**) Absorbance at 282 nm of released Pdop fragments as a function of time and pH (3, 5, 6.8, and 9). (**c**) Photographs of the Pdop fragment solutions at pH 3, 5, 6.8, and 9 after 3 h. (**d**) UV–Vis absorption spectra of Pdop fragments released from Pdop particles at pH 6.8 over 120 h. (**e**) Absorbance at 282 nm of released Pdop fragments at pH 6.8 over 120 h.

The Pdop particles used in this study were synthesized following a previously reported method [12,15]. Pdop fragments can be released from Pdop particles into the aqueous solution at pH 6.8 (Figure 3a). The release solution, initially a light brown color, gradually darkened over time. Figure 3a shows the time-dependent UV–Vis spectra of this solution. A strong absorbance peak near 282 nm, attributed to Pdop, appeared and increased in intensity over a period of 3 h, confirming the sustained release of Pdop fragments from the particles. Pdop itself exhibits broad absorption in the visible region due to its melanin-like structure [36]. However, when Pdop undergoes degradation, the released fragments are likely smaller molecules containing specific chromophores, such as catechol and indole derivatives [37,38]. Previous studies on dopamine monomers and oligomers indicate a characteristic absorption peak around 275–285 nm [37,38]. Furthermore, our experimental results demonstrate a direct correlation between the amount of released fragments and the absorbance at 282 nm. This suggests that 282 nm is a suitable indicator for tracking the release process. To investigate the effect of pH on the release of Pdop fragments, Pdop release tests were conducted at pH 3, 5, 6.8, and 9. Similar phenomena to those observed at pH 6.8 were also observed at these pH values. However, compared to pH 6.8, a significantly larger amount of Pdop fragments was released, with a 4.3-fold increase at pH 3, a 1.6-fold increase at pH 5, and a 1.1-fold increase at pH 9 (Figure 3b and Figure S3). Among the release solutions, the solution at pH 3 exhibited the darkest color (Figure 3c). These results indicate that Pdop is more unstable in acidic conditions and slightly less stable in basic conditions compared to neutral conditions. In other words, these results show that Pdop is more prone to degradation under acidic conditions, but the release of Pdop fragments occurs across a wide range of pH values. A notable observation was the burst release of Pdop fragments from the Pdop particles within 10 min across a wide range of pH values (Figure 3b and Figure S3). Thus, to investigate the long-term stability of Pdop particles, release tests were conducted at pH 6.8 over a 120 h period (Figure 3d). The release of Pdop fragments steadily increases for 120 h after the initial burst release at 10 min, suggesting that Pdop particles undergo continuous fragmentation even under neutral conditions (Figure 3e). These results suggest that without an effective method to prevent the release of Pdop fragments, the structural degradation of Pdop particles will accelerate in almost all pH ranges, ultimately leading to their complete disintegration.

**Figure 4:**
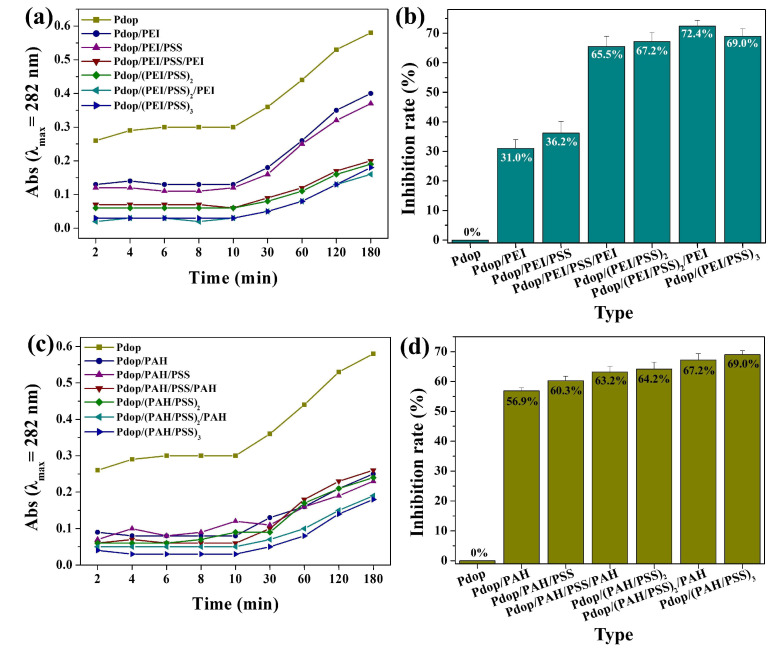
Evaluation of PEM coating effect on Pdop fragment release and inhibition rates. (**a**) Absorbance at 282 nm of released Pdop fragments from Pdop/(PEI/PSS)_3_ particles as a function of coating layer number and time. (**b**) Corresponding inhibition rates calculated from the data in (**a**). (**c**) Absorbance at 282 nm of released Pdop fragments from Pdop/(PAH/PSS)_3_ particles as a function of coating layer number and time. (**d**) Corresponding inhibition rates calculated from the data in (**c**).

To investigate the effect of coating layer number on the stability of Pdop particles, PEMs consisting of PEI and PSS were coated onto the Pdop particles. The release of Pdop fragments from the Pdop particles decreased as the number of PEM-coating layers increased (Figure 4a and Figure S4). Notably, a significant increase in inhibition rates was observed upon the coating of odd-numbered layers (first, third, and fifth PEI coating), reaching 31%, 29.3%, and 5.2%, respectively (Figure 4b). In contrast, the average inhibition rate increased by only 1.2% upon the coating of even-numbered layers (second, fourth, and sixth PSS coating) (Figure 4a,b). These results suggest that a positively charged PE layer, such as PEI, is more effective than a negatively charged PE layer, such as PSS, in preventing the release of Pdop fragments. This observation can be attributed to the enhanced electrostatic attraction between positively charged PEI and negatively charged Pdop fragments. To further validate our hypothesis, PEMs consisting of PAH and PSS were coated onto the Pdop particles and evaluated. As observed in Figure 4a,b, Pdop release was suppressed as the number of PEM layers increased (Figure 4c,d and Figure S5). Moreover, this phenomenon was more pronounced in odd-numbered layers (first, third, and fifth PAH coating) (Figure 4c,d). These findings further support our hypothesis that negatively charged Pdop fragments can be effectively captured by positively charged PEI or PAH through electrostatic attraction, thereby preventing the release of Pdop fragments.

**Figure 5:**
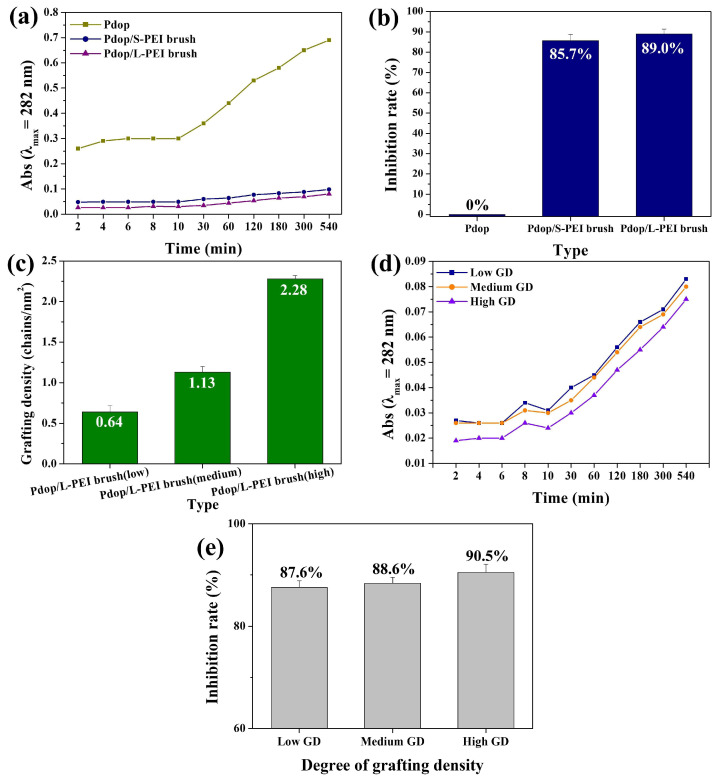
Influence of PEI brush chain length and grafting density on Pdop fragment release and inhibition rates. (**a**) Absorbance at 282 nm of released Pdop fragments from Pdop particles grafted with short-chain PEI (S-PEI) and long-chain PEI (L-PEI) brushes as a function of time. (**b**) Corresponding inhibition rates calculated from the data in (**a**). (**c**) Pdop/L-PEI brush particles with varying grafting densities (low, medium, and high). (**d**) Absorbance at 282 nm of released Pdop fragments from Pdop/L-PEI brush particles with varying grafting densities (low, medium, and high) as a function of time. (**e**) Corresponding inhibition rates calculated from the data in (**d**).

Given that grafted PE offers a higher density of available active sites compared to coated PE [35], the PE-grafting approach was employed to prevent the release of Pdop fragments. XPS measurements were performed to confirm PEI brush grafting onto the Pdop particles. The N 1s spectrum of the Pdop particles exhibited four components: –N= (398 eV), –NH- (398.6 eV), –NH_2_ (400.6 eV), and –N-C- (402 eV) (Figure S6). Following PEI grafting, the -NH- and –NH_2_ peak intensity increased relative to the other components (Figure S6), indicating successful PEI grafting onto the Pdop surface via Michael addition between catechol groups and primary amines [8,11]. To investigate the effect of varying PE chain length on the stability of Pdop particles, short (S)-chained PEI (S-PEI, MW 25,000 Da) and long (L)-chained PEI (L-PEI, MW 750,000 Da) brushes were grafted onto the surface of Pdop particles. Both S and L-chained grafting approaches effectively prevented the release of Pdop fragments from the Pdop particles (Figure 5a,b and Figure S7). Notably, both grafting methods exhibited significantly higher inhibition rates compared to the PEM coating methods (Figure 5a,b and Figure 4b,d). Furthermore, L-chained grafting demonstrated a higher inhibition rate than S-chained grafting (Figure 5a,b). These results demonstrate that the PE-grafting method is more effective than PEM coating in preventing the release of Pdop fragments, with L-chained PE exhibiting superior inhibition ability compared to S-chained PE. Figure 5a further highlights a significant difference between the two approaches. While the PEM-coating method effectively suppressed the release of Pdop fragments, it exhibited limited ability to prevent the initial burst release observed within the first 10 min. In contrast, the PE-grafting method effectively mitigated this burst release because grafted PE offers a higher density of available active sites to capture Pdop fragments compared to coated PEM (Figure 5a). To investigate the effect of grafting density on the stability of Pdop particles, L-chained PEI brushes with varying grafting densities were grafted onto the surface of Pdop particles. Three types of L-PEI brushes were prepared, exhibiting low, medium, and high grafting densities. The grafting density (σ) of the L-PEI brush (chains/nm^2^) was calculated using Equation (1):

σ = (Γ N_A_)/M_W_ (1)

where N_A_ is Avogadro’s number, and M_W_ is the molecular weight of PEI. The surface concentration (Γ) of the PEI brush film can be calculated using Equation (2):

Γ = d ρ_o_ (2)

where d is the thickness, and ρ_o_ is the density of the PEI brush film (assumed to be 1.03 g/cm^3^).

The L-PEI brushes exhibited low, medium, and high grafting densities of 0.64, 1.13, and 2.28 chains/nm^2^, respectively (Figure 5c). The inhibition rates increased with increasing grafting density of PEI chains, suggesting that a higher grafting density of PEI chains provides more effective capture of Pdop fragments (Figure 5d,e and Figure S8).

**Figure 6:**
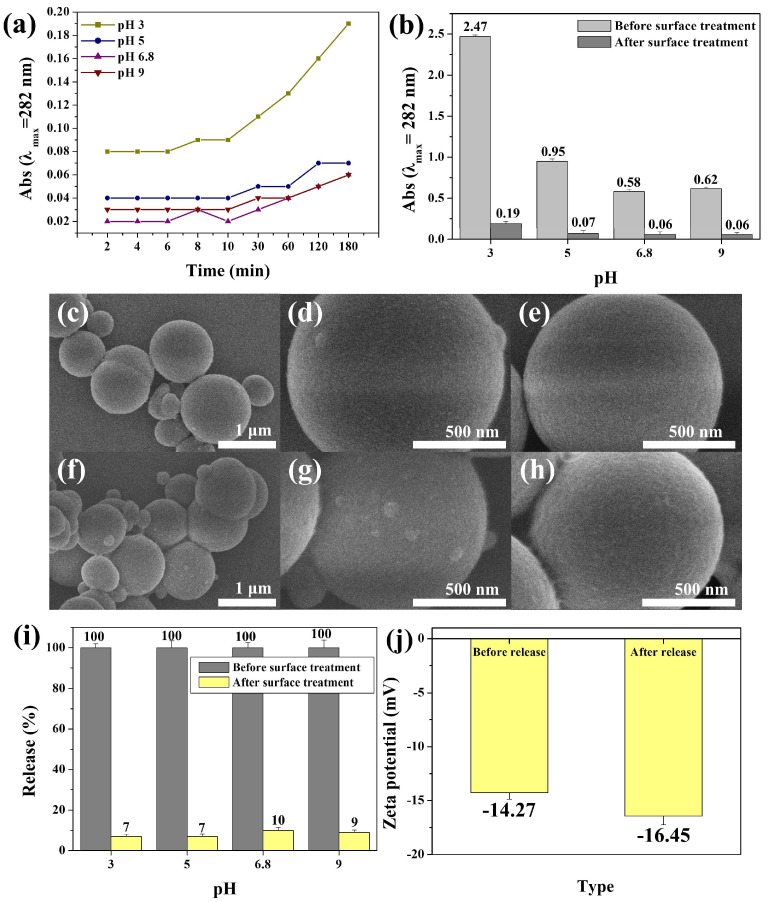
Assessment of pH stability and morphological changes in bare Pdop and Pdop/L-PEI brush particles after fragment release. (**a**) Absorbance at 282 nm of released Pdop fragments from Pdop/L-PEI brush particles as a function of time and pH (3, 5, 6.8, and 9). (**b**) Comparison of absorbance at 282 nm of released Pdop fragments from bare Pdop particles and Pdop/L-PEI brush particles as a function of time and pH (3, 5, 6.8, and 9). (**c**) Corresponding Pdop fragment release percentages calculated from the data in (**b**). (**d**–**f**) SEM images of bare Pdop particles after the release of Pdop fragments. (**g**–**i**) SEM images of Pdop/L-PEI brush particles after the release of Pdop fragments. (**j**) Zeta potential of Pdop/L-PEI brush particles before and after the release of Pdop fragments.

To investigate the stability of L-PEI (2.28 chains/nm^2^)-grafted Pdop particles, Pdop release tests were conducted across a wide range of pH values. While exhibiting similar release profiles to bare Pdop particles (Figure 6a, Figure S9, and Figure 3b), the L-PEI-grafted Pdop particles demonstrated significantly reduced fragment release. Compared to bare Pdop particles, the L-PEI-grafted particles showed a substantial decrease in fragment release at all tested pH values: 7% at pH 3, 7% at pH 5, 10% at pH 6.8, and 9% at pH 9 (Figure 6b,c). Compared to bare Pdop particles, the L-PEI-grafted Pdop particles (2.28 chains/nm^2^) demonstrated a high level of inhibition, with rates ranging from 90% to 93% across a wide range of pH values (Figure 6b,c). After Pdop release, while bare Pdop particles exhibited a smooth surface morphology (Figure 6d–f), the Pdop/PEI brush particles displayed a relatively rough surface (Figure 6g–i). A similar phenomenon was observed in the UV–Vis data. The absorbance of Pdop particles in water (pH 6.8) decreased after 5 h, suggesting the release of Pdop fragments (Figure S10). However, under the same conditions, the absorbance of Pdop/PEI brush particles did not decrease significantly, indicating that the released Pdop fragments were readsorbed onto the surface of the Pdop/PEI brush particles (Figure S10). This observation suggests that the PEI brush effectively captured released Pdop fragments. Zeta-potential measurements further supported this hypothesis. The surface charge of the Pdop/PEI brush shifted from −14.27 mV to −16.45 mV after Pdop release (Figure 6j). This negative shift in surface charge is attributed to the adsorption of released Pdop fragments by the PEI brush, as the formation of PEI/Pdop fragment complexes imparted a more negative charge to the Pdop particle surface. Our proposed surface engineering approach has the potential to enable the synthesis and application of stable Pdop-based materials across a wide pH range.

## 4. Conclusions

This study successfully demonstrated the effectiveness of surface engineering strategies in enhancing the stability and mitigating fragmentation of Pdop particles. Two approaches were investigated: (1) PEM coating and (2) PE brush grafting. PEM coating with alternating layers of positively and negatively charged PEs demonstrated varying degrees of inhibition, with odd-numbered layers exhibiting significantly higher inhibition rates. PE brush grafting, particularly with long-chain PEI, proved to be highly effective in suppressing Pdop fragment release, including the initial burst release. Furthermore, L-PEI grafting with higher densities exhibited enhanced inhibition rates. The L-PEI-grafted particles demonstrated exceptional stability across a wide pH range, with inhibition rates exceeding 90% in most cases. These findings provide valuable insights into the design and fabrication of stable Pdop-based materials with enhanced performance and expanded applications in various fields, including biomedicine, catalysis, energy, and environmental remediation. Future research will focus on exploring different types of PE brushes, optimizing the grafting conditions, and evaluating the long-term stability of the modified Pdop particles in real-world environments.

## Data Availability

The original contributions presented in this study are included in the article/Supplementary Material. Further inquiries can be directed to the corresponding authors.

## Supplementary Materials

The following supporting information can be downloaded at https://www.mdpi.com/article/10.3390/polym17091209/s1: Figure S1: FT-IR spectra of Pdop/(PEI/PSS)_3_ and Pdop/(PAH/PSS)_3_ multilayers; Figure S2: Zeta-potential measurements of Pdop/(PEI/PSS)_3_ and Pdop/(PAH/PSS)_3_ multilayers; Figure S3: UV–Vis absorption spectra of Pdop fragments released from Pdop particles at pH 3, pH 5, and pH 9 as a function of time; Figure S4: UV–Vis absorption spectra of Pdop fragments released from Pdop/PEI, Pdop/PEI/PSS, Pdop/(PEI/PSS)/PEI, Pdop/(PEI/PSS)_2_, Pdop/(PEI/PSS)_2_/PEI, and Pdop/(PEI/PSS)_3_ particles at pH 6.8 as a function of time; Figure S5: UV–Vis absorption spectra of Pdop fragments released from Pdop/PAH, Pdop/PAH/PSS, Pdop/(PAH/PSS)/PAH, Pdop/(PAH/PSS)_2_, Pdop/(PAH/PSS)_2_/PAH, and Pdop/(PAH/PSS)_3_ particles at pH 6.8 as a function of time; Figure S6: XPS spectra of Pdop particles and Pdop/PEI brush particles. High-resolution N 1s spectra of Pdop particles before and after PEI grafting, with peak deconvolution. Percentage (%) surface area of N-containing functional groups (-N=, -NH-, -NH₂, and -N-C-) observed in Pdop and Pdop/PEI brush samples; Figure S7: UV–Vis absorption spectra of Pdop fragments released from Pdop/Short-PEI brush and Pdop/Long-PEI brush particles at pH 6.8 as a function of time; Figure S8: UV–Vis absorption spectra of Pdop fragments released from Pdop particles grafted with L-PEI brushes of low, medium, and high grafting densities at pH 6.8 as a function of time; Figure S9: UV–Vis absorption spectra of Pdop fragments released from Pdop particles grafted with L-PEI brushes of high grafting density at pH 3, pH 5, pH 6.8, and (d) pH 9 as a function of time. Figure S10. UV-Vis absorption spectra of (a) Pdop particles and (b) Pdop/PEI brush particles before and after dispersion in water at pH 6.8 for 5 h.
