# Supplementary material for: Surface Modification of Polydopamine Particles with Polyethyleneimine Brushes for Enhanced Stability and Reduced Fragmentation
Source: Polymers (Basel). 2025 Apr 28;17(9):1209. doi: 10.3390/polym17091209 (PMC12073504; doi:10.3390/polym17091209)
Supplement: Supplementary file 1 [file polymers-17-01209-s001.zip › polymers-3561363-supplementary.pdf]

Supporting Information

Surface Modification of Polydopamine Particles with Polyethyleneimine Brushes for Enhanced Stability and Reduced Fragmentation

Su Hyeon Son[1,†], Eun Jin Kim[1,†], Hye Young Koo[2,], and Won San Choi[1,*]*

[1]Department of Chemical and Biological Engineering, Hanbat National University, 125 Dongseodaero, Yuseong-gu, Daejeon 305-719, Republic of Korea; E-mail: shujh19@naver.com; kimej106@naver.com ; choiws@hanbat.ac.kr, [2]Functional Composite Materials Research Center, Jeonbuk Institute of Advanced Composite Materials, Korea Institute of Science and Technology (KIST), 92 Chudong-ro, Bongdong-eup, Wanju-gun, 55324, Republic of Korea; E-mail: koohy@kist.re.kr

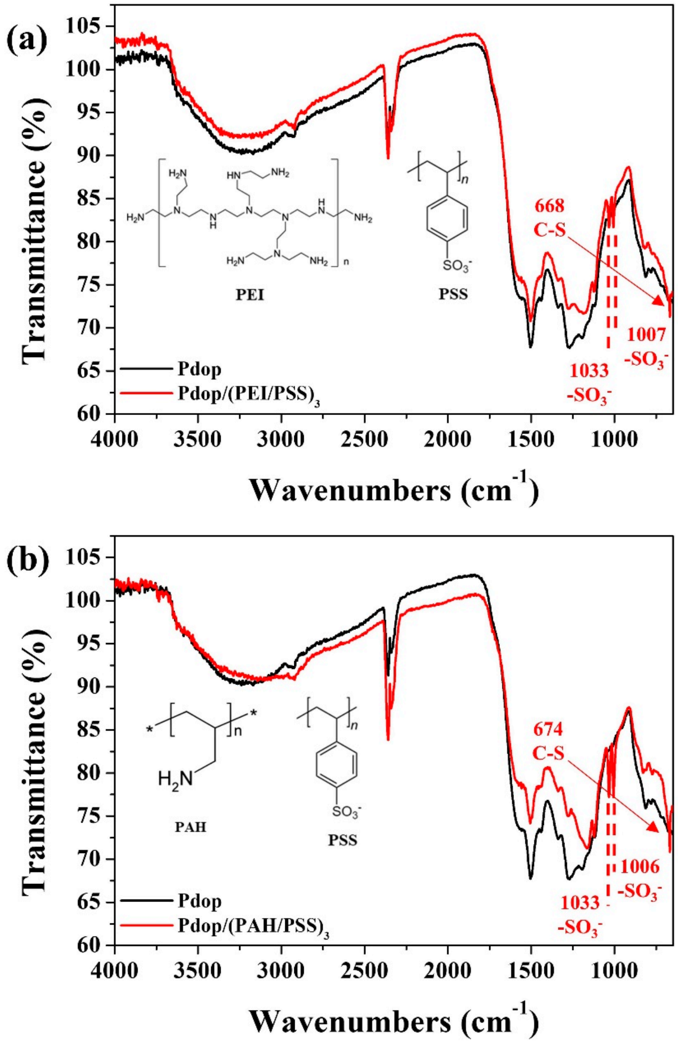

Figure S1. FT-IR spectra of (a) Pdop/(PEI/PSS)$_3$ and (b) Pdop/(PAH/PSS)$_3$ multilayers.

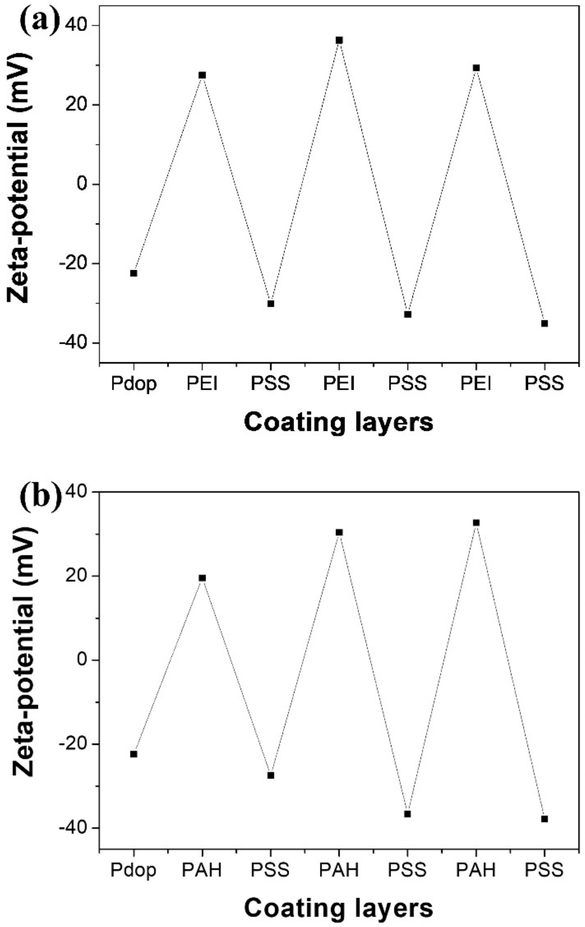

Figure S2. Zeta-potential measurements of (a) Pdop/$(PEI/PSS)_3$ and (b) Pdop/$(PAH/PSS)_3$ multilayers.

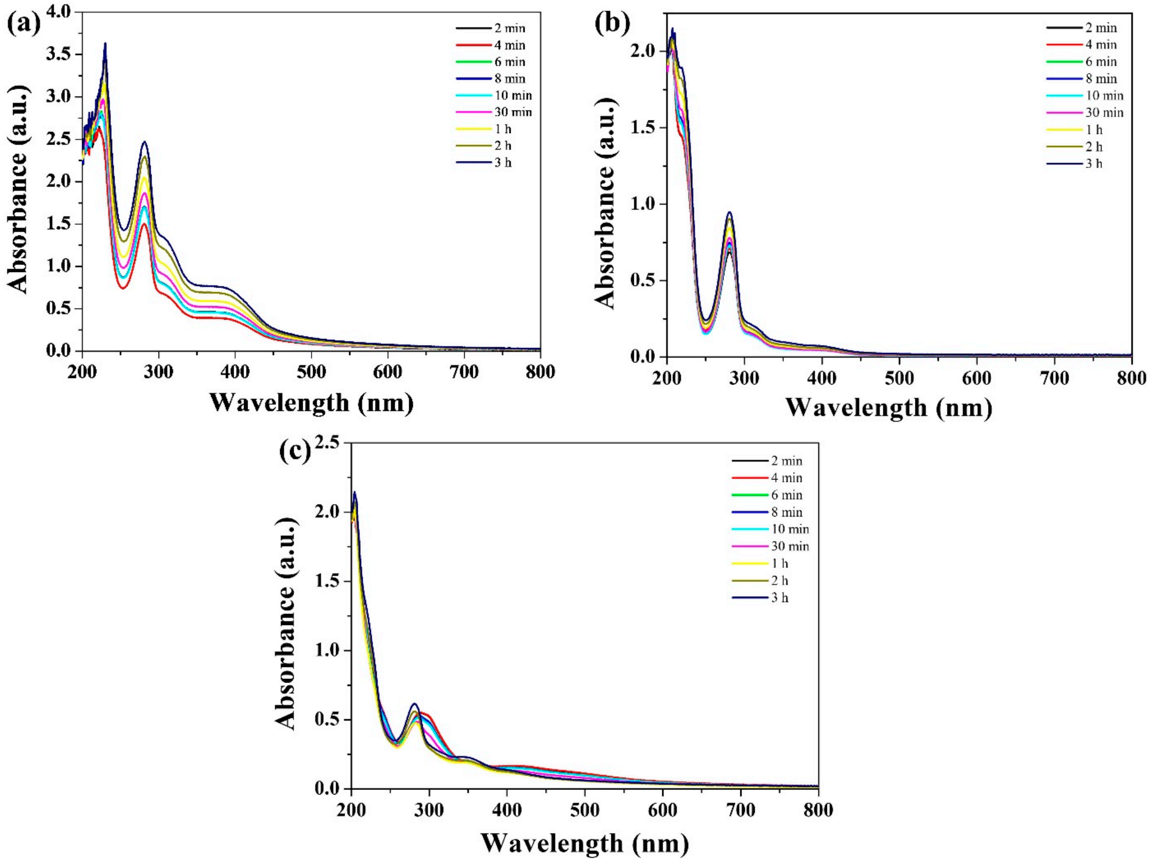

Figure S3. UV-Vis absorption spectra of Pdop fragments released from Pdop particles at (a) pH 3, (b) pH 5, and (c) pH 9 as a function of time.

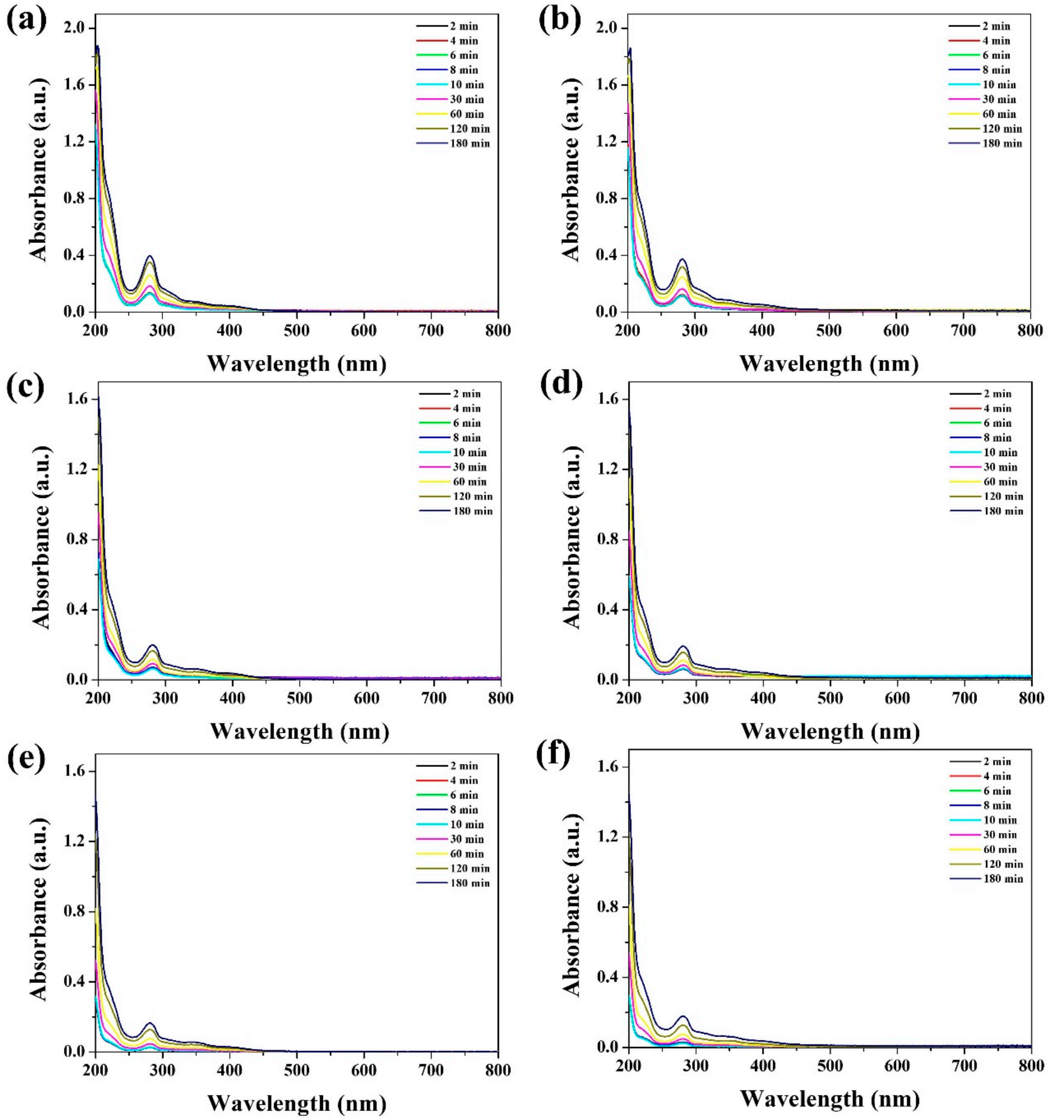

Figure S4. UV-Vis absorption spectra of Pdop fragments released from (a) Pdop/PEI, (b) Pdop/PEI/PSS, (c) Pdop/(PEI/PSS)/PEI, (d) Pdop/(PEI/PSS)$_2$, (e) Pdop/(PEI/PSS)$_2$/PEI, and (f) Pdop/(PEI/PSS)$_3$ particles at pH 6.8 as a function of time.

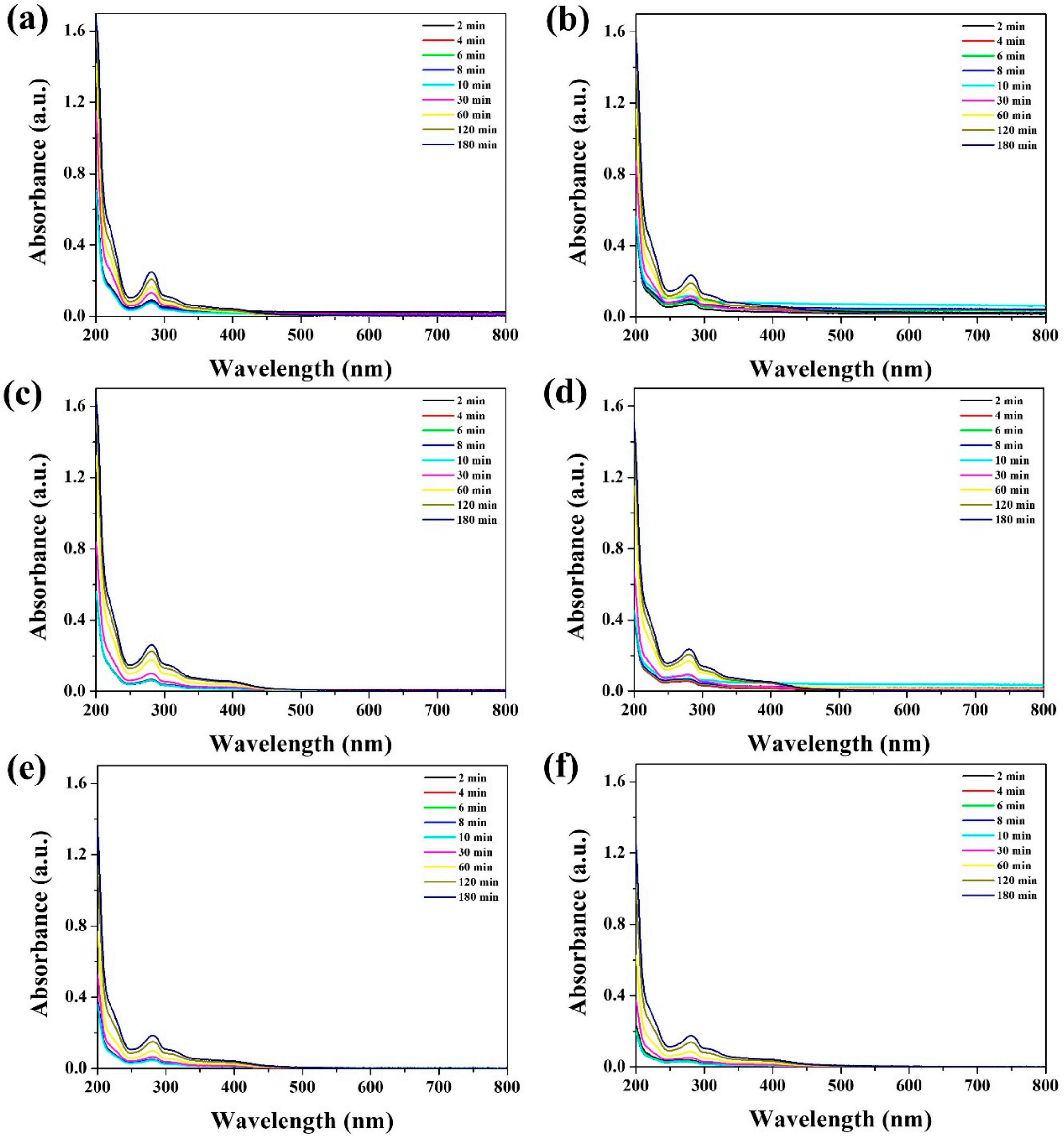

Figure S5. UV-Vis absorption spectra of Pdop fragments released from (a) Pdop/PAH, (b) Pdop/PAH/PSS, (c) Pdop/(PAH/PSS)/PAH, (d) Pdop/(PAH/PSS)$_2$, (e) Pdop/(PAH/PSS)$_2$/PAH, and (f) Pdop/(PAH/PSS)$_3$ particles at pH 6.8 as a function of time.

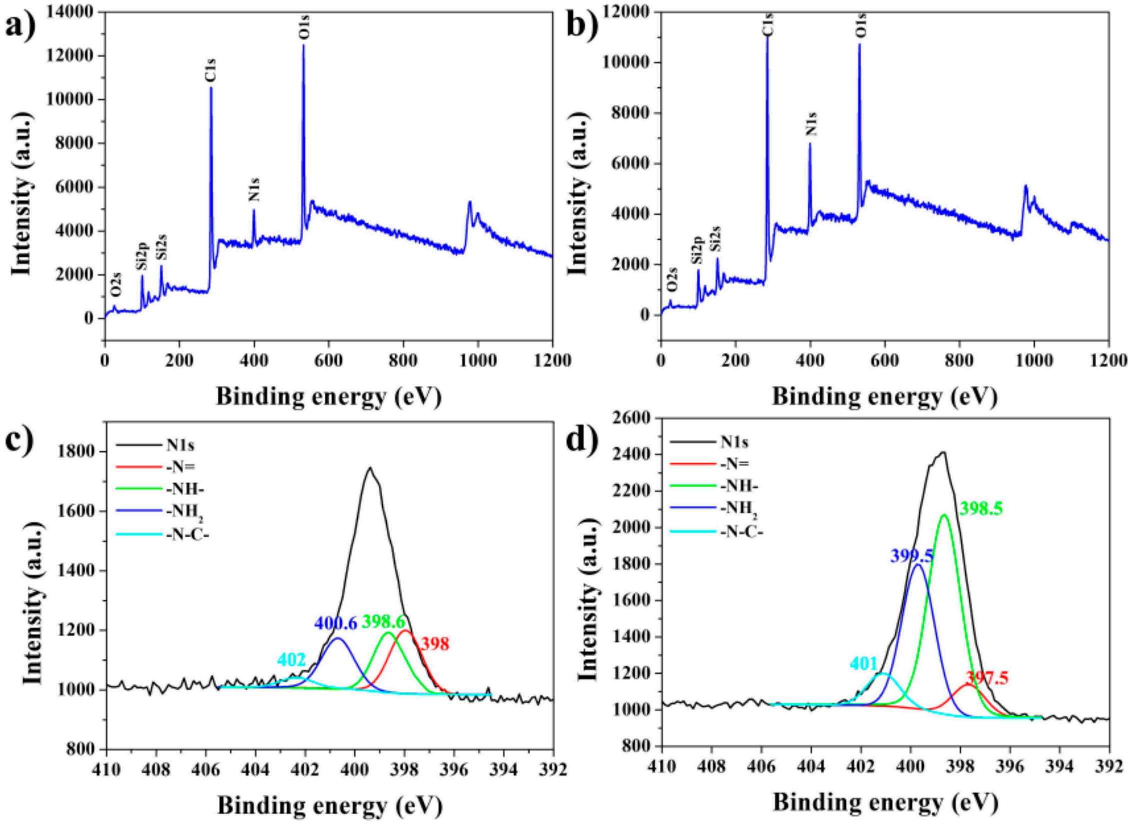

e)

	%Area	
	**Pdop**	**Pdop/PEI brush**
**-N=**	**32.02**	**8.10**
**-NH-**	**30.83**	**49.63**
**$-NH_2$**	**30.94**	**34.46**
**-N-C-**	**6.21**	**7.81**

Figure S6. X-ray photoelectron spectroscopy (XPS) spectra of (a, c) Pdop particles and (b, d) Pdop/PEI brush particles. High-resolution N 1s spectra of Pdop particles (c) before and (d) after PEI grafting, with peak deconvolution. (e) Percentage (%) surface area of N-containing functional groups (-N=, -NH-, $-NH_2$, and -N-C-) observed in Pdop and Pdop/PEI brush samples.

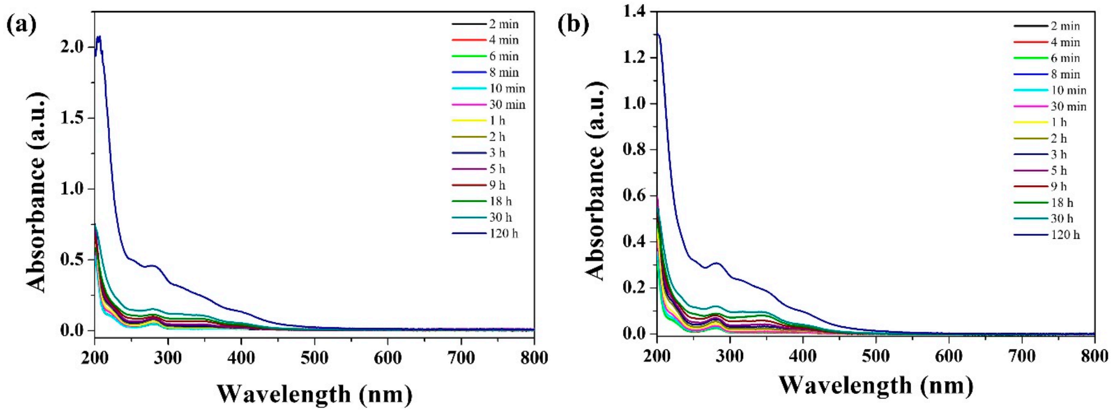

Figure S7. UV-Vis absorption spectra of Pdop fragments released from (a) Pdop/Short-PEI brush and (b) Pdop/Long-PEI brush particles at pH 6.8 as a function of time.

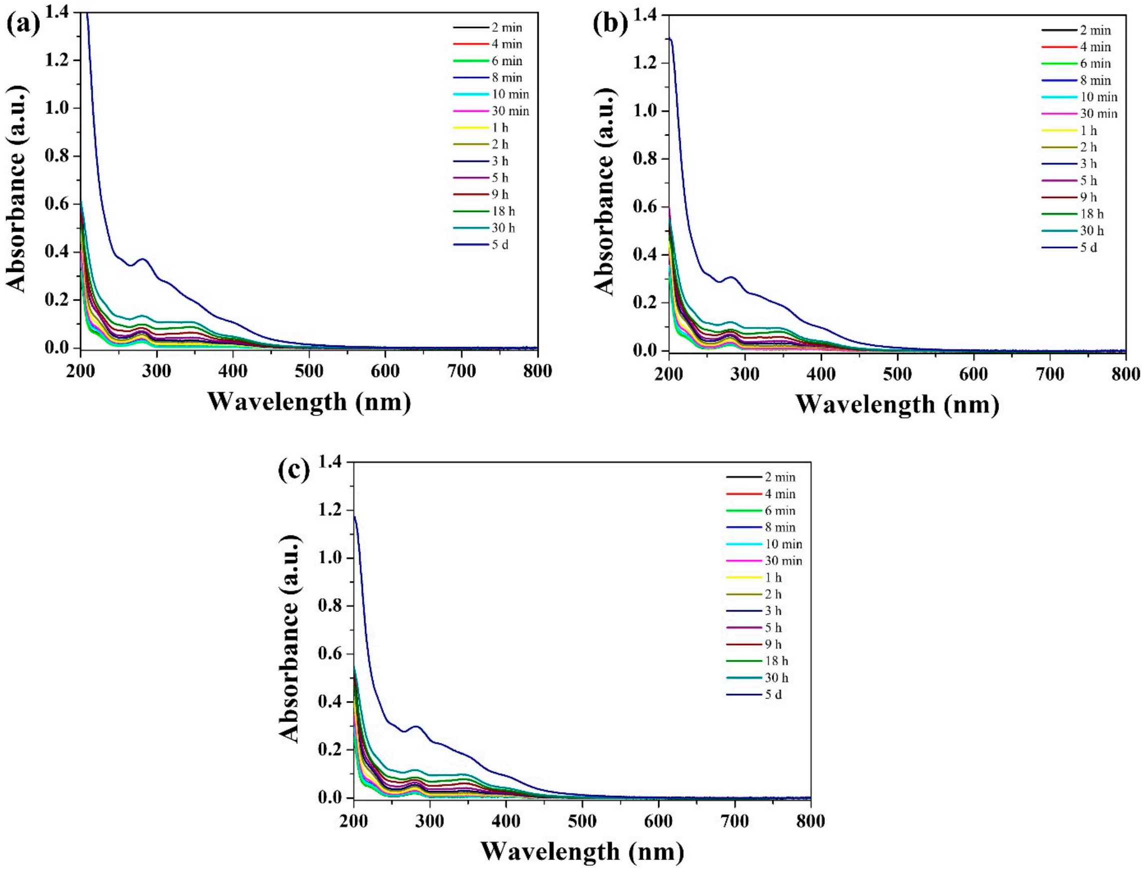

Figure S8. UV-Vis absorption spectra of Pdop fragments released from Pdop particles grafted with L-PEI brushes of (a) low, (b) medium, and (c) high grafting densities at pH 6.8 as a function of time.

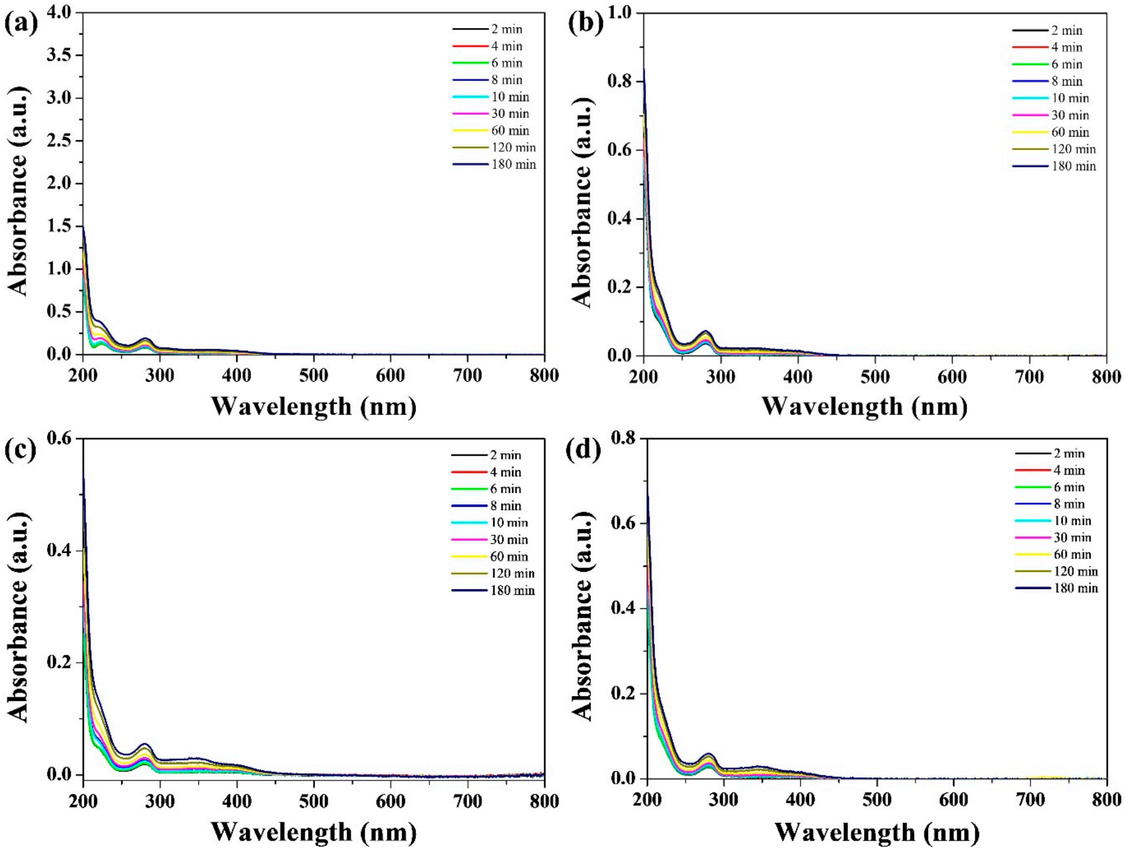

Figure S9. UV-Vis absorption spectra of Pdop fragments released from Pdop particles grafted with L-PEI brushes of high grafting density at (a) pH 3, (b) pH 5, (c) pH 6.8, and (d) pH 9 as a function of time.

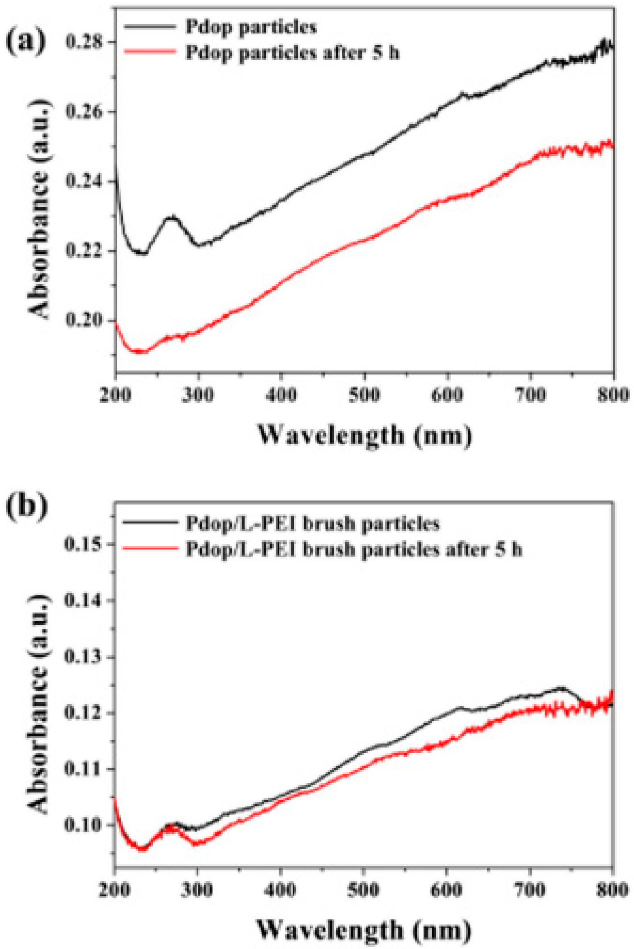

Figure S10. UV-Vis absorption spectra of (a) Pdop particles and (b) Pdop/PEI brush particles before and after dispersion in water at pH 6.8 for 5 h.
